# Multimorbidity patterns and risk of hospitalisation in children: A population cohort study of 3.6 million children in England, with illustrative examples from childhood cancer survivors

**DOI:** 10.1016/j.lanepe.2022.100433

**Published:** 2022-06-30

**Authors:** Sheng-Chia Chung, Stefanie Mueller, Katherine Green, Wai Hoong Chang, Darren Hargrave, Alvina G. Lai

**Affiliations:** a Institute of Health Informatics, University College London, London, UK; b Great Ormond Street Hospital, London, UK; c University College London Great Ormond Street Institute of Child Health, London, UK

**Keywords:** Childhood cancer survivors, Multimorbidity, Hospitalisation risk, Electronic health records, Child health, Health disparity

## Abstract

**Background:**

Population-level estimates of hospitalisation risk in children are currently limited. The study aims to characterise morbidity patterns in all children, focusing on childhood cancer survivors versus children without cancer.

**Methods:**

Employing hospital records of children aged <19 years between 1997 to 2018 in England, we characterised morbidity patterns in childhood cancer survivors compared with children without cancer. The follow-up began on the 5^th^ anniversary of the index hospitalisation and the primary outcome was the incidence of comorbidities.

**Findings:**

We identified 3,559,439 eligible participants having 12,740,666 hospital admissions, with a mean age at study entry of 11.2 years. We identified 32,221 patients who survived for at least 5 years since their initial cancer diagnosis. During the follow-up period and within the whole population of 3.6 million children, the leading conditions for admission were (i) metabolic, endocrine, digestive renal and genitourinary conditions (84,749, 2.5%), (ii) neurological (35,833, 1.0%) and (iii) musculoskeletal or skin conditions (23,574, 0.7%), fever, acute respiratory and sepsis (22,604, 0.7%). Stratified analyses revealed that females and children from socioeconomically deprived areas had a higher cumulative incidence for morbidities requiring hospitalisation (*p* < 0.001). At baseline (5 years after the initial cancer diagnosis or initial hospitalisation for survivors and population comparisons, respectively), cancer survivors experienced a higher prevalence of individual conditions and multimorbidity (≥ 2 morbidities) compared with children without cancer. Cox regression analyses showed that survivors had at least a 4-fold increase in the risk of hospitalisation for conditions such as chronic eye conditions (hazard ration (HR):4.0, 95% confidence interval (CI): 3.5-4.7), fever requiring hospitalisation (HR: 4.4, 95% CI: 3.8-5.0), subsequent neoplasms (HR: 5.7, 95% CI:5.0-6.5), immunological disorders (HR: 6.5, 95% CI:4.5-9.3) and metabolic conditions (HR: 7.1, 95% CI:5.9-8.5).

**Interpretation:**

The overall morbidity burden among children was low in general; however, childhood cancer survivors experienced a higher prevalence and subsequent risk of hospitalisation for a range of morbidities. Targeted policies may be required to promote awareness on health vulnerabilities and gender disparity and to improve advocacy for healthcare in deprived communities.

**Funding:**

Wellcome Trust, National Institute for Health Research (NIHR) University College London Hospitals Biomedical Research Centre, NIHR Great Ormond Street Hospital Biomedical Research Centre and Academy of Medical Sciences. The funders of the study had no role in study design, data collection, data analysis, data interpretation, or writing of the report.

**Research in context**

**Evidence before this study**

We searched between May, 2001, and May 2021 PubMed for clinical studies included keywords of combining “morbidity”, “children”, “electronic health records”, “nationwide” in the title; and clinical studies included “comorbidity” and “children” in the title, “childhood cancer survivor” or “paediatric cancer survivor” in the title and “electronic health records” or “EHR” in the text. Relevant reports and studies cited in the identified studies were also reviewed. Whilst previous studies provide insights into of different late effects of childhood cancer, comprehensive, population-based evidence on the incidence of morbidity and risk of hospitalisation for acute and chronic conditions in children (childhood cancer survivors and children without cancer) was absent.

**Added value of this study**

Our findings add to the literature a more contemporary comorbidity profile in all children and robust estimates of the variations of hospitalisation risk over time in at-risk groups. By investigating all admissions of 32,221 childhood cancer survivors and 3.5 million children without cancer across England, we found the incident morbidity risk was higher in girls and children from areas of higher socioeconomic deprivation, especially in children without cancer. Compared to children without cancer, childhood cancer survivors had a more than 3-fold increased risk of hospitalised infection, sepsis, anaemia and other blood disorders, chronic eye conditions, hospitalised fever, subsequent neoplasms, immunological disorders and metabolic comorbidity.

**Implications of all the available evidence**

Our study identified conditions that are common in children and in childhood cancer survivors, which may assist in the needs-based assessment of long-term surveillance and resource allocation. We envision results from this study may contribute to improvements in the support provided to children experiencing both severe health conditions and gender or socioeconomic health disparity, and the development of late effects service guidelines and follow-up care plans for childhood cancer survivors.

Alt-text: Unlabelled box

## Introduction

Diseases in childhood and adolescence have a long-term impact on subsequent health during adulthood, with dire consequences on communities and societies.^1^^,^^2^ Linked populational electronic health records (EHRs) enable detailed investigations on risk factors and the burden of specific childhood diseases,^3^, ^4^, ^5^ with additional granularity by age group^6^ and demographic factors that may have previously been underpowered using datasets of insufficient sample size. Comprehensive, population-based evidence on the incidence of morbidity and risk of hospitalisation for acute and chronic conditions in children and adolescents have been currently lacking.

Children who survived cancer have a higher risk of subsequent morbidity. Previous studies in Scandinavia,^7^ UK,^8^ Netherlands,^9^ and US^10^^,^^11^ showed that childhood cancer survivors not only have an early onset of subsequent morbidity,^12^ but also an increased risk of hospitalisation.^7^, ^8^, ^9^^,^^13^ As improvements in cancer treatment and supportive care over the past three decades have resulted in over 80% of children surviving beyond five years, cancer survivors are facing a unique set of health challenges that may extend into adulthood.^14^^,^^15^ Multimorbidity is also common among childhood cancer survivors.^16^^,^^17^ Given these increased health risks, clinical guidelines have recommended the development of a care plan that details follow-up and sequelae monitoring arrangements for children and young adults who have had cancer.^18^ However, thorough investigations on the patterns and extent of potential morbidities and subsequent risk of hospitalisation have been limited to small scale studies despite the need for an evidence-based and risk-stratified care plan that prioritises individuals who are most at risk.

The publicly funded healthcare system in the UK (the National Health Service (NHS)) provides a universal healthcare coverage for all; individuals with private health insurance consisted of only 10% of the UK population.^19^ Therefore, this study employs a representative dataset of hospital admission records for 3.6 million children in England to investigate morbidity patterns and hospitalisation in children, while drawing comparisons between childhood cancer survivors and children who have not had cancer. Specific objectives include: 1) to characterise morbidity patterns in all children and childhood cancer survivors who were alive on the 5^th^ year of the index admission, 2) to analyse the risk of hospitalisation for different types of comorbidities in childhood cancer survivors compared with children without cancer and 3) to estimate the differences in hospitalisation risks by age, sex, cancer type and socioeconomic deprivation. Our findings add to the literature a more contemporary comorbidity profile for all children, providing robust estimates (generated from a large sample size) on the variations of hospitalisation risk over time, highlighting at-risk groups. Our work may inform the planning and commissioning of health services for children and adolescents and help prioritise the allocation of diagnostic and therapeutic resources for disease management based on available data-informed evidence.

## Methods

### Dataset

We employed the NHS Hospital Episode Statistics (HES) dataset that was collected and available for the study from 1997 to 2018. HES is a record-level dataset collected by the National Health service on all admissions to NHS hospitals in England as part of patient care and support.^20^ HES data contain patient, clinical and administrative details for in-patients, outpatients and accident and emergency admissions. Each patient record in HES is defined as a Finished Consultant Episode (FCE) that corresponded to the care delivered under a consultant. A patient may have more than one FCEs from hospital admission to discharge. Each record in HES had up to 20 diagnostic codes recorded using the 10th revision of the International Classification of Diseases (ICD-10) and up to 12 interventions and procedural codes recorded using the Office of Population Censuses and Surveys Classification of Interventions and Procedures version 4 (OPCS-4), with the first entry during the study period indicating the primary diagnosis or intent of the episode respectively.^21^ We identified all hospital in-patient admissions (Admitted Patient Care [APC]) and accident and emergency (AE) admissions for study participants. Permission to use de-identified data from Hospital Episode Statistics was granted by NHS Digital (DARS–NIC-06527). This work received ethics approval from the Health Research Authority Research Ethics Committee and the London Camden & Kings Cross Committee (18/LO/0010). The study is in accordance with the REporting of studies Conducted using Observational Routinely-collected health Data (RECORD) guidelines.^22^

### Study design and population

The target population of this longitudinal cohort study consisted of all children, including cancer survivors (who survived for at least five years since their initial cancer diagnosis) and children without cancer as the comparator population. All individuals aged below 19 years at the initial hospital admission or AE admissions were included. We defined incident cancer diagnoses as patients having any records of ICD-10 C00-97, and children with cancer were identified as individuals having their first cancer diagnosis under the age of 19 years. Given that we only had ICD-10 data available, cancer types were defined according to ICD-10 classification (Supplementary Table S1). Childhood cancer survivors were defined as those who have survived for at least five years from the first date of their primary cancer diagnosis. Follow-up for comorbidities requiring inpatient hospitalisation began five years after the date of cancer diagnosis in childhood cancer survivors (baseline). We considered children without cancer as those who were free of any cancer diagnosis up until the age of 19 years. Follow-up for comorbidities in children without cancer began five years after the date of their first hospital record. Children with invalid entry dates or who exited the cohort before reaching the 5th anniversary of the index admission (entry date) were excluded in from the analyses. Follow-up ended on death or the date of administrative censoring (November 2018), whichever occurred first.

### Outcomes

The primary outcome was the incidence of inpatient hospitalisation by comorbidity. We considered 26 comorbidities in 10 diagnostic categories, adapted from the comprehensive list of comorbidities reported previously^23^ (supplementary table S2). These included: A) mental health and behavioural disorders, including 1.substance abuse, 2.self-harm/other mental health problem, 3.Behavioural/development disorders; B)/4. Subsequent neoplasm, C) immunological and blood disorders: 5.Immunological disorders, 6.anaemia and other blood disorders, D) chronic infections: 7. tuberculosis and other infection; E) respiratory conditions: 8.asthma and chronic lower respiratory disease, 9.other respiratory; F) metabolic, endocrine, digestive, renal, genitourinary (GU) conditions: 10.diabetes, 11.metabolic, 12.digestive, 13.renal or genitourinary, 14.metabolic or gastrointestinal injuries or other conditions, G) musculoskeletal or skin conditions: 15. musculoskeletal or connective tissue, 16.skeletal injuries or amputations, 17.chronic skin disorders; H) neurological conditions: 18.epilepsy, 19.injuries of brain, nerves, eyes or ears, 20.chronic eye conditions, 21.chronic ear conditions, 22.other neurological; I) cardiovascular disease: 23.other cardiovascular; J) fever, acute respiratory and sepsis: 24. fever requiring hospitalisation, 25.acute respiratory, 26.sepsis diagnoses during the follow-up period (Supplementary Table S2). The initial hospital admission for each condition was used to define the occurrence of the condition. Multimorbidity at study entry was defined as having two or more chronic comorbidities, in addition to cancer, at the beginning of the follow-up. We also reported the proportion of individuals with multimorbidity during the study period among individuals who did not have a history of multimorbidity at baseline.

### Other covariates

We have included in the study the explanatory variables of age (continuous and categorised into categories of 0-4, 5-9, 10-14 and 15-18 years old), sex and index of multiple deprivation^24^ quintiles. Among survivors, we investigate additional subgroups of cancer type (Supplementary Table S2).

### Statistical analyses

The prevalence of chronic conditions and multimorbidity at the beginning of follow-up (i.e., baseline, the fifth anniversary of the index admission) were categorised into groups of childhood cancer survivors and children without cancer. Data were summarised according to patient demographics and patient age (by 5-year age categories) for survivors at initial cancer diagnosis at baseline. We summarised the cumulative incidence of comorbidities during follow-up by sex and socioeconomic deprivation status^24^ by cancer type in survivors. In time-to-event analyses (supplementary Figure S2 and Section 1), we used Cox proportional hazards model to compare the age, sex and deprivation-adjusted incidence between childhood cancer survivors and children without cancer for each of the comorbidity categories. We applied Cox models to estimate cause-specific hazard where competing events were treated as censored observations.^25^^,^^26^ Proportional hazards assumption violations were tested for a zero slope in the scaled Schoenfeld residuals. All analyses were performed using SAS (version 9·4) and R (version 3·2·3).

## Results

The study cohort included 3,559,439 eligible children and 12,740,666 hospital admissions (Figure S1). The study excluded 1,690,523 children who exited the cohort before reaching their entry dates. The study follow-up extends into early to mid-adulthood. 32,221 individuals survived 5 years or longer post-cancer diagnosis. At the end of the study, the mean age was 20 years (standard deviation (SD): 8.2 years) for all children and 23 (SD: 7.7 years) for survivors. The median duration of follow-up in survivors was 8.7 years (range 0–17.1 years), during which we observed 1,156,238 hospital admissions among cancer survivors. Children without a cancer diagnosis (i.e., the comparator population) consisted of 3,527,218 individuals, with a median follow-up of 8.5 years (SD: 5.0 years).

### Prevalence of morbidities at study entry

The distributions of conditions, which were identified from hospital admissions, at the beginning of follow-up in the study population were summarised in Table 1. The prevalence of conditions by hospital admissions among all children ranged from 0.2% (95% confidence interval (CI) for prevalence:(0.2-0.2%)) in chronic infection to 9.7% (CI: 9.6-9.7%) for fever, acute respiratory conditions, or sepsis. Childhood cancer survivors had a higher prevalence of health conditions at study entry, ranging from 0.5% (CI: 0.5-0.6%) for mental health conditions to 37.5% (CI: 37-38%) for immunological and blood disorders. When considering individuals with two or more non-cancer comorbidities (multimorbidity), the prevalence was 33.6% (CI: 33-34%) in survivors versus 2.0% (CI: 1.9-2.0%) in children without cancer. We observed similar prevalence in females and males for comorbid conditions at baseline in all children and both the cancer survivors and children without cancer (Table 1). In survivors, children who were diagnosed with cancer between the ages of 0 to 4 years had an increased prevalence of multimorbidity (48.2%, (CI: 47.3- 49.2%)) at study entry compared with those diagnosed between the ages of 15 to 18 years (17.3%, (CI: 16.5-18.1%)). Additionally, when considering the prevalence of each condition separately, the trend remained similar where children diagnosed between the ages of 0 to 4 years had the highest prevalence in immunological or blood disorders, chronic infections, metabolic, endocrine, digestive renal and genitourinary conditions, cardiovascular conditions and fever, acute infection and sepsis than comparisons (Table 1).

**Table 1:** Prevalence of comorbid conditions at baseline in the study population by demographic characteristics.

Study population	N	Types of comorbidity									
		1) Mental health/behavioural	2) Immuno/blood disorders	3) Chronic infections	4) Respiratory	5) Metabolic/endocrine/digestive/renal/ GI	6) Musculo-skeletal/skin	7) Neurological	8) Cardiovascular	9) Fever/acute respiratory/sepsis	With 2 or more types of comorbidities
All children	3559439	27002 (0.8%)	40516 (1.1%)	6716 (0.2%)	80434 (2.3%)	159592 (4.5%)	31542 (0.9%)	94731 (2.7%)	25173 (0.7%)	343617 (9.7%)	79670 (2.2%)
All childhood cancer survivors	32221	174 (0.5%)	12086 (37.5%)	233 (0.7%)	623 (1.9%)	3185 (9.9%)	1176 (3.6%)	2976 (9.2%)	4928 (15.3%)	9485 (29.4%)	10831 (33.6%)
Population comparisons	3527218	26828 (0.8%)	28430 (0.8%)	6483 (0.2%)	79811 (2.3%)	156407 (4.4%)	30366 (0.9%)	91755 (2.6%)	20245 (0.6%)	334132 (9.5%)	68839 (2%)
Boy - survivors	17626	96 (0.5%)	6705 (38%)	142 (0.8%)	352 (2%)	1729 (9.8%)	652 (3.7%)	1675 (9.5%)	2780 (15.8%)	5316 (30.2%)	6015 (34.1%)
Girl- survivors	14595	78 (0.5%)	5381 (36.9%)	91 (0.6%)	271 (1.9%)	1456 (10%)	524 (3.6%)	1301 (8.9%)	2148 (14.7%)	4169 (28.6%)	4816 (33%)
Boy - comparisons	1843166	12285 (0.7%)	14664 (0.8%)	3595 (0.2%)	48735 (2.6%)	67023 (3.6%)	13416 (0.7%)	50261 (2.7%)	10721 (0.6%)	185612 (10.1%)	36353 (2%)
Girl- comparisons	1684052	14543 (0.9%)	13766 (0.8%)	2888 (0.2%)	31076 (1.8%)	89384 (5.3%)	16950 (1%)	41494 (2.5%)	9524 (0.6%)	148520 (8.8%)	32486 (1.9%)
Least deprived quintile - survivors	6311	34 (0.5%)	2320 (36.8%)	38 (0.6%)	103 (1.6%)	603 (9.6%)	214 (3.4%)	559 (8.9%)	941 (14.9%)	1857 (29.4%)	2090 (33.1%)
Most deprived quintile - survivors	6773	46 (0.7%)	2679 (39.6%)	54 (0.8%)	152 (2.2%)	673 (9.9%)	252 (3.7%)	662 (9.8%)	1079 (15.9%)	2028 (29.9%)	2345 (34.6%)
Least deprived quintile - comparisons	555265	4423 (0.8%)	3877 (0.7%)	883 (0.2%)	10644 (1.9%)	24858 (4.5%)	5183 (0.9%)	14326 (2.6%)	3675 (0.7%)	48139 (8.7%)	9371 (1.7%)
Most deprived quintile - comparisons	885931	6546 (0.7%)	7646 (0.9%)	1759 (0.2%)	22363 (2.5%)	39147 (4.4%)	7249 (0.8%)	23033 (2.6%)	4651 (0.5%)	91287 (10.3%)	19299 (2.2%)
**Among survivors**, by age at initial cancer diagnosis											
0-4 years	10795	22 (0.2%)	5396 (50%)	93 (0.9%)	239 (2.2%)	1354 (12.5%)	244 (2.3%)	1123 (10.4%)	2427 (22.5%)	4876 (45.2%)	5204 (48.2%)
5-9 years	6443	35 (0.5%)	2713 (42.1%)	42 (0.7%)	129 (2%)	556 (8.6%)	295 (4.6%)	686 (10.6%)	958 (14.9%)	2037 (31.6%)	2294 (35.6%)
10-14 years	6855	55 (0.8%)	2347 (34.2%)	53 (0.8%)	132 (1.9%)	600 (8.8%)	361 (5.3%)	612 (8.9%)	861 (12.6%)	1485 (21.7%)	1929 (28.1%)
15-18 years	8128	62 (0.8%)	1630 (20.1%)	45 (0.6%)	123 (1.5%)	675 (8.3%)	276 (3.4%)	555 (6.8%)	682 (8.4%)	1087 (13.4%)	1404 (17.3%)

*A person can be in multiple comorbidity groups and multi-comorbidities if applicable. Baseline: 5th anniversary of the index hospital admission in survivors and non-cancer comparisons. Multimorbidity at baseline defined as 2 or more morbidities.

### Incidence of subsequent hospitalisation

During the follow-up period and within the whole population of 3.6 million children, 84,749 (2.5%) were admitted to hospital for metabolic, endocrine, digestive renal and genitourinary conditions, 35,833 (1.0%) for neurological conditions, 23,574 (0.7%) for musculoskeletal or skin conditions, 22,604 (0.7%) for fever, acute respiratory and sepsis, 18,177 (0.5%) for respiratory conditions, 16,576 (0.5%) for conditions related to mental health and behaviour, 13,812 (0.4%) for neoplasms, 11,771 (0.3%) for cardiovascular conditions, 8,239 (0.2%) for immunological or blood disorders, and 1,059 (0.03%) for chronic infections (Figure S3). When comparing cancer survivors with children who have not had cancer, the cumulative incidence of hospital admissions during follow-up were higher as follows: mental health and behaviour conditions (0.5% vs. 0.5%), immunological or blood disorders (1.2% vs. 0.2%), chronic infections (0.1% vs. 0.03%), respiratory conditions (0.7% vs. 0.5%), metabolic, endocrine, digestive renal and genitourinary conditions (3.8% vs. 2.5%), musculoskeletal or skin conditions (1.3% vs. 0.7%), neurological conditions (2.6% vs. 1.0%), cardiovascular conditions (1.2% vs. 0.3%), fever, acute respiratory and sepsis (2.0% vs 0.7%) and neoplasm (4.2% vs. 0.4%) (*p*all <0.001 except *p* = 0.6 for mental health and behaviour) (Figure S3). The cumulative incidence of multimorbidity during follow-up was 5.3% in survivors and 1.3% in children without cancer (*p* < 0.001).

**Figure 1:**
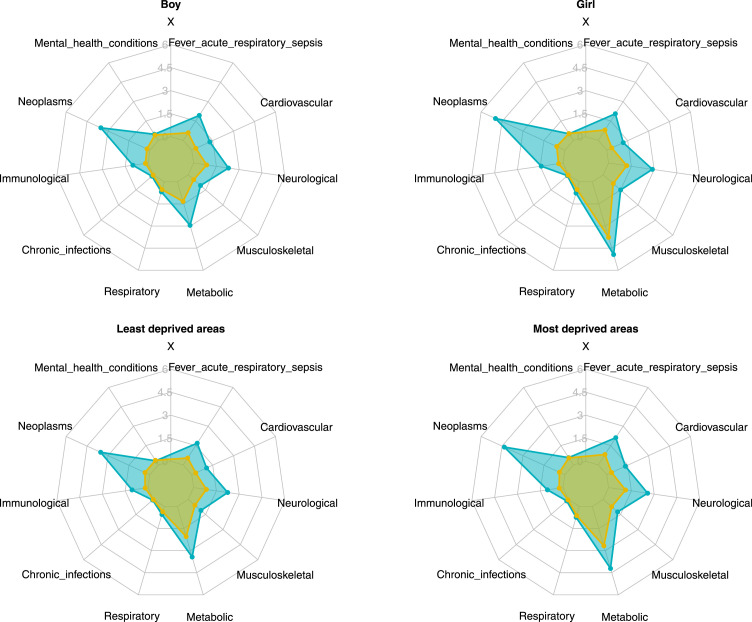
Cumulative incidence of hospital admissions of condition categories by sex and socioeconomic deprivation status. Each of the condition category is displayed as a spoke on the radar plot. Blue larger polygon: survivors (greater cumulative incidence); yellow smaller polygon: comparators (lower cumulative incidence).

The cumulative incidence of metabolic conditions was higher (*p* < 0.001) in girls than in boys, survivors or children without cancer alike (Figure 1, Supplementary Table S4). Childhood cancer surviving girls had a higher incidence of subsequent neoplasm hospital admission than boys (*p* = 0.009). Children living in neighbourhoods with higher socioeconomic deprivation had a greater incidence in all conditions (*p* all <0.001). Among survivors, higher socioeconomic deprivation was associated with a greater cumulative incidence of mental health and behaviour (*p* = 0.04) and fever, acute respiratory and sepsis (*p* = 0.04).

**Figure 2:**
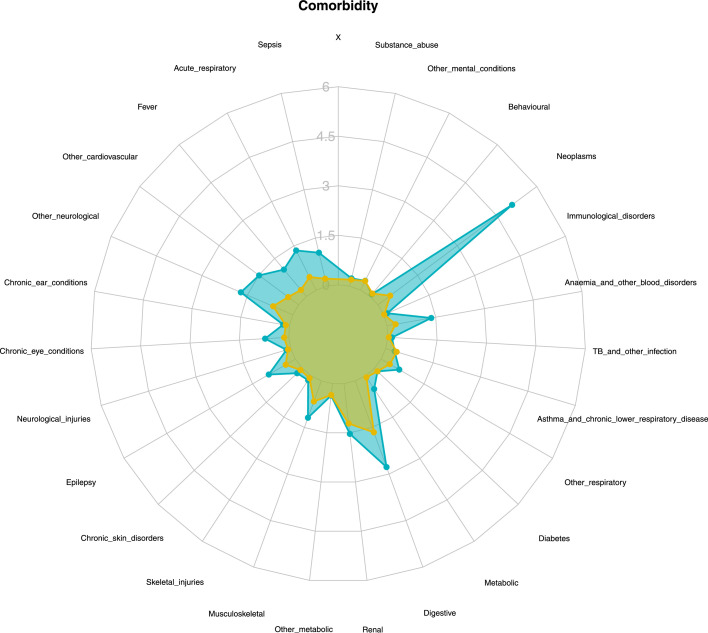
Cumulative incidence of comorbidities requiring hospitalisation. Each of the comorbidities is displayed as a spoke on the radar plot. Blue larger polygon: survivors (greater cumulative incidence); yellow smaller polygon: comparators (lower cumulative incidence).

Figure 2 summarises the cumulative incidence for subsequent hospitalisation of the 26 detailed comorbidities investigated in the study. Cumulative incidences among childhood cancer survivors were subsequent neoplasms (cumulative incidence: 4.2%, 95% CI:3.7%,4.8%), digestive (2.3% (2.1%,2.5%)), other neurological (1.4%(1.3%,1.6%)), renal or genitourinary (1.3% (1.2%,1.4%)) and other cardiovascular (1.2% (1.1%,1.4%)). In comparison, the leading incident conditions were digestive (1.4% (1.4%,1.4%)), renal or genitourinary (1.0%(1.0%,1.0%)), musculoskeletal or connective tissue (0.55% (0.5%,0.6%)), other neurological (0.54% (0.53%,0.55%)), acute respiratory (0.37% (0.36%,0.38%)) in children who did not have cancer.

**Figure 3:**
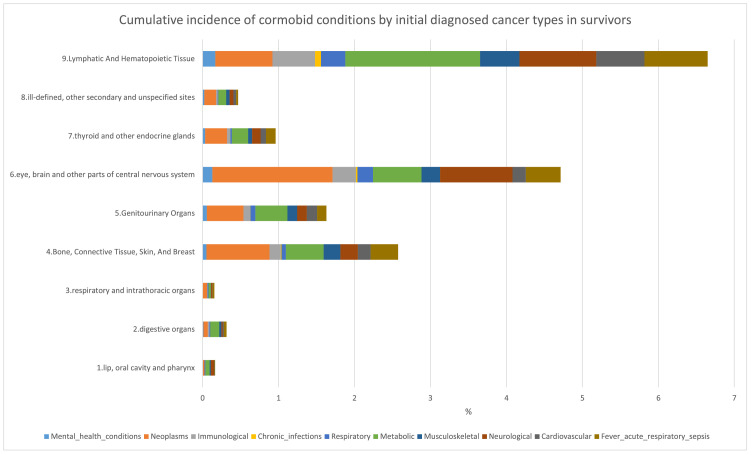
Distribution of cumulative incidence of conditions requiring hospitalisation in survivors of different initial cancer types. *The denominator was all childhood cancer survivors free from the condition, and the numerator was survivors with the index cancer developing the incident comorbid condition (for example, survivors with lymphatic cancer developing a particular incident comorbidity).

In childhood cancer survivors, after stratifying by their initial cancer types, we found that the prevalence of multimorbidity at baseline was the highest among children with haematological cancers (14.5%), which is important as leukaemia represents the commonest cancer in childhood (Figure 3). During follow-up, more survivors with an index cancer admission of the eye, brain and other parts of central nervous system, and lymphatic and hematopoietic tissue developed comorbid conditions during follow-up (Figure 3). Stratification by the nine cancer types revealed a high occurrence of incident comorbidity in survivors with an index cancer diagnosis at thyroid and other endocrine glands (Supplementary figure S4). There were differences in cumulative incidence for subsequent hospitalisation when comparing across cancer types. For example, children who were initially diagnosed with cancer in digestive organs were more likely to develop metabolic or digestive-related comorbidities during follow-up (Supplementary figure S4). Children who were initially diagnosed with cancer of the central nervous system, eye or brain had a higher incidence of neurological diseases during follow-up.

### Childhood cancer survivors had a higher risk of hospitalisation for selected comorbidities during follow-up

After accounting for age, sex difference and socioeconomic deprivation, survivors had a significantly higher risk of hospital admission for immunological or blood disorders, chronic infections, metabolic, endocrine, digestive renal and genitourinary conditions, musculoskeletal or skin disorders, neurological conditions, cardiovascular conditions, respiratory, fever, acute infection and sepsis and neoplasms (*p* all < 0.001) (Supplementary figure S5).

**Figure 4:**
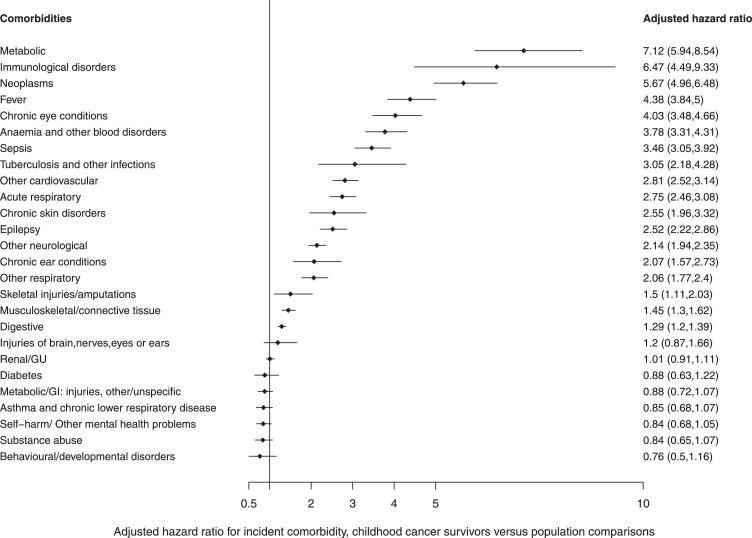
Age- and sex-adjusted Cox regression analyses for risk of hospital admissions of comorbidities by cancer survivors compared with children without cancer as the reference. *The hazard ratios are based on incidence.

From the adjusted Cox regression analyses, we observed that childhood cancer survivors had a 2 to 3-fold increased risk of hospitalisation for 7 conditions: other respiratory (adjusted hazard ratio: 2.1 (95% confidence interval: 1.8-2.4)), chronic ear conditions (2.1 (1.6-2.7)), other neurological (2.1 (1.9-2.4)), chronic skin disorders (2.6 (2.0-3.3)), epilepsy (2.5 (2.2-2.9)), acute respiratory (2.8(2.5-3.1)), other cardiovascular (2.8 (2.5-3.1)). Survivors had a more than 3-fold increased risk of hospitalisation for a further 8 comorbidities: tuberculosis and other infection (3.1 (2.2-4.3)), sepsis (3.5 (3.1-3.9)), anaemia and other blood disorders (3.8 (3.3-4.3)), chronic eye conditions (4.0(3.5-4.7)), fever requiring hospitalisation (4.4 (3.8-5.0)), subsequent neoplasms (5.7 (5.0-6.5)), immunological disorders (6.5 (4.5-9.3)), metabolic comorbidity (7.1 (5.9-8.5)) (Figure 4).

## Discussion

Our study reports a comprehensive investigation of chronic health conditions in 3.6 million children, including childhood cancer survivors and children without cancer using population hospital records. Individuals were followed-up through early and mid-adulthood. We studied 10 condition categories consisting of 26 specific comorbidities. Despite the low incidence of comorbidity requiring hospitalisation among children, childhood cancer survivors experienced a 2 to 3-fold increased risk of hospitalisation for morbidities including respiratory injuries, chronic ear conditions, other neurological, other respiratory, chronic skin disorders, epilepsy, acute respiratory, other cardiovascular, and survivors had a more than 3-fold increased risk of infection, sepsis, anaemia and other blood disorders, chronic eye conditions, fever, subsequent neoplasms, immunological disorders and metabolic comorbidity. Cumulative morbidity incidence in children differed by sex and socioeconomic deprivation and by index cancer type in survivors. Our results highlight the importance for children health services to address the gender and socioeconomic health disparity and the value of multidisciplinary clinical support to identify and monitor adverse effects experienced by childhood cancer survivors to improve long-term health outcomes and wellbeing.

Clinical guidelines^27^, ^28^, ^29^ for long term management of childhood cancer survivors recommend regular monitoring for subsequent malignancy, fertility complications, cardiac conditions, bone mineral density, ototoxicity, thyroid dysfunction, endocrinological disorder, metabolic syndrome, cognitive, neuropsychological, and psychosocial conditions – as corroborated by our findings. Additionally, based on detailed comorbidity analyses, we observed that childhood cancer survivors had a higher risk of hospitalisation for complications in the respiratory system (chronic or acute), chronic disorders in skin or eyes, fever, infection and sepsis. These conditions could have arisen as a result of the primary cancer or metastatic disease, treatment toxicity or infection of skins, ocular and pulmonary function.^16^^,^^30^, ^31^, ^32^, ^33^ Others have shown that sepsis, fever and infection are potential complications in paediatric oncology patients^34^^,^^35^; survivors may have impaired immune systems,^36^ which increases the susceptibility to pathogens. Our results showed the risk remained elevated after five years after the initial cancer diagnosis. Long-term management of paediatric cancer patients continues into adulthood, thus, our findings may inform evidence-based surveillance and prevention measures for skin cancer,^37^ ocular^30^ or pulmonary dysfunction,^38^ and infection^36^ in these patients.

We observed that among children without cancer, girls had a higher baseline prevalence of hospitalisation for mental health, metabolic, musculoskeletal or skin conditions. During follow-up, girls also had a higher incidence of all comorbidities except for chronic infection and respiratory conditions. Higher morbidity burden in women has been reported previously,^39^^,^^40^ especially for mental, neurological, and musculoskeletal disorders^39^ and our study showed that the gender discrepancy in morbidity risk could potentially start in childhood. For cancer survivors, at baseline, the prevalence of morbidities was comparable for either sex. However, during follow-up, girls had a higher cumulative incidence of conditions, suggesting that the long‐term impact of cancer may disproportionately affect females. We observed that female survivors were more likely to be hospitalised for subsequent cancer and metabolic or musculoskeletal conditions. Our findings were corroborated by other studies reporting that females are more likely to have a higher risk of long-term adverse outcomes^9^^,^^17^^,^^41^ and treatment-associated cardiomyopathy, heart failure.^42^^,^^43^ Females survivors were also at greater risk of subsequent malignancy, due to a higher risk of subsequent breast^16^ and thyroid cancer.^44^ Sex-specific risks of long-term complications of survivors are reviewed elsewhere to highlight potential applications of sex-specific cancer therapy.^44^

While socioeconomic deprivation is associated with a higher prevalence of multimorbidity in adults,^45^^,^^46^ the impact of deprivation on multimorbidity in children is poorly understood. We found that in both cancer survivors and children without cancer, the prevalence of individual comorbidities and multimorbidity at baseline was higher in children living in deprived areas. Likewise, during follow-up, children from more deprived areas had a higher incidence of hospital admissions. A meta-analysis of the relationship between childhood chronic conditions and socioeconomic status revealed that the most common disabling chronic conditions were associated with high deprivation.^47^ The impact of disparity on the prevalence of chronic conditions could also be explained by other factors such as substance abuse, educational attainment, nutrition and healthcare access.^48^ Our findings on a higher incidence of mental health conditions in survivors living in areas with high socioeconomic deprivation suggest a greater need for psychosocial support for survivors and their carers.

We observed that one in three survivors had experienced two or more conditions on the 5^th^ anniversary of the initial cancer diagnosis. The high prevalence of multimorbidity was reported previously^17^ and may be explained by cancer-related or therapy-related toxicities and stressors.^16^ Our results showed that risks of comorbidities among survivors varied by type of cancer, and we observed associations between the index cancer site and the location of the sequelae. Treatment-induced organ system dysfunction has been reported as a major risk factor contributing to subsequent morbidity in childhood cancer survivors,^9^^,^^13^^,^^16^^,^^49^^,^^50^ and previous studies have reported childhood cancer survivors had an increased risk of hospitalisation for conditions such as endocrine, nutritional and metabolic diseases, subsequent cancer, skin, musculoskeletal, autoimmune (e.g., anaemia, diabetes, thyroid dysfunction and arthropathy associated disorder), cardiovascular, circulatory, or neurological diseases (e.g., epilepsy or neurocognitive impairment).^9^^,^^13^^,^^49^, ^50^, ^51^, ^52^ The greater likelihood of multimorbidity observed in the present study may increase the infection risk and worsening prognosis, for example, in the COVID-19 pandemic, cancer survivors were found more likely to develop severe outcomes after SARS-CoV-2 infection.^53^^,^^54^

### Implications for clinicians, policymakers, patients and carers

To the best of our knowledge, this study is the first to demonstrate variations in hospitalisation incidence and risk over time for 3.6 million children using population health records. Risks of varying comorbid conditions differed by sex and socioeconomic deprivation. Our results have implications for health policy as girls have greater vulnerability to diseases and children and carers from disadvantaged backgrounds could experience a higher risk of developing comorbidities. However, perceptions of health vulnerability do not always translate to practices of healthy behaviours.^55^ Potential barriers to implementation include sustained risky behaviours (e.g., smoking), inequalities brought on by socioeconomic differences (e.g., poverty) that influence access to optimal care, and the availability of collaborative care delivery and care plan integrating different clinical specialties in the health system,^16^ taking into account the sex difference in disease risk factors, social factors and health behaviours.^40^ Evidence-based interventions, such as comprehensive approaches integrating children's services, from health and social care to education, environment, and housing, may help mitigate the adverse impact of childhood risk factor exposures. Effective innovations such as involving staff from social care, mental health, and community-based initiatives, working alongside clinicians in primary or secondary care^56^ could improve the support provided to children experiencing both severe health conditions and deprivation.

Among childhood cancer survivors, our study identified conditions that are common, which may assist in the needs-based assessment of long-term surveillance and resource allocation. Additionally, we envision that results from this study may contribute to the development of late effects service guidelines and follow-up care plans for cancer survivors in the following ways: 1) provision of evidence-based hospitalisation risk information by comorbidity type to advise health authorities on populations that are at higher risks for screening or counselling, 2) informing the identification of potential risk groups for more or less frequent follow-up and hospital visits depending on evidence-based risk of survivor morbidity, 3) empowering carers and young adult survivors to take control of long-term risks by providing personalised risk information to help them identify health problems early, encourage reporting of symptoms and participation in screening (e.g., for early onset of cardiovascular conditions), 4) development of evidence-based guidance for dietary and lifestyle advice to reduce the prevalence of metabolic syndrome and obesity in survivors, and 5) lowering the threshold for referral of suspected cancer given our observation that survivors have increased risk of subsequent hospitalisation due to cancer. Health data and datasets on wider determinants of health should be used to monitor and understand the efficacy and effectiveness of such intervention for evidence-aid decision making.^56^ In the current COVID-19 pandemic, it is important to strengthen prevention measures, such as nonpharmaceutical interventions and vaccination, among childhood cancer survivors and their close contacts due to their greater vulnerability to SARS-Cov-2 infection and severe outcomes.

### Strength and limitations

There are three major strengths of this study. Firstly, the study uses the national population dataset includes 3.6 million children and 12.7 million hospital admissions. It not only provides an important estimation of the reference comorbidity profile in all children but enables a detailed investigation of comorbidities in childhood cancer survivors. One of the advantages of national coverage is that it may improve case ascertainment and the total cohort size, thus with the benefit of reduced potential biases caused by missing, non-respondent^8^^,^^51^ or targeting of selected data. Long-term follow-up has allowed us to provide robust estimates of the variations of hospitalisation risk over time. Secondly, we provided the nationwide estimates of comorbidity prevalence and risk of hospitalisation in childhood cancer survivors compared with other children without cancer. This has important health, wellbeing and care resource applications given that cancer is the commonest cause of non-traumatic death in childhood. Thirdly, we have analysed all cancer comorbidity patterns to underpin estimates of hospitalisation risks. With a median follow-up duration of 9 years, we were able to investigate conditions that have a delayed presentation in childhood cancer survivors.

We acknowledge the limitation of using secondary care records, which exclude children without hospital contact and may unintentionally bias the reporting of prevalence and incidence of comorbidities which may be higher than in the general population. We cannot completely rule out the possibility of children with their initial cancer diagnosis prior to the data accessible to us. However, as repeated hospital admissions are commonly required for cancer management, such patients were likely to be included in the study. Our study did not include analyses by cancer treatment, as complete information on cancer therapy and details such as dosage or field are not available. We acknowledge that long-term therapy-related toxicities are common and may result in functional declines^57^ and excess deaths due to subsequent neoplasms, pulmonary and cardiovascular diseases.^58^ Future studies could focus on investigating morbidity patterns associated and differences in hospitalisation risks associated with cancer treatment. Unlike in the National Cancer Registration and Analysis Service dataset, information on cancer stage and grade were also unavailable. Nonetheless, cancer registry data does not allow the analysis of comorbidities in children without cancer and lacks comprehensive information on diagnosis afforded by population records. We acknowledge that this study does not capture conditions that are managed in primary care. Childhood cancers are classified based on tumour site and tumour morphology, with greater emphasis on morphology. Classification employs the International Classification for Childhood Cancer (ICCC) with site and morphology coded in International Classification of Diseases for Oncology (ICD-O). However, since secondary care records are coded in ICD-10, we were unable to analyse the risk of hospitalisation based on the more accurate ICCC categories. Estimations of the prevalence of leukaemia and lymphoma by ICD-10 subcategories in the study were consistent with the figures reported by the National Cancer Intelligence Network,^59^ suggesting the validity of the study results given the differences in coding systems.

### Future research

We have identified several areas for further research. There is a dearth of evidence on long-term adverse effects from clinical trials of cancer drugs in children. Since the launch of the European Paediatric Medicine Regulation, there has been a considerable impact on improving the efficacy and safety of medicines in children.^60^^,^^61^ As new empirical data on treatment becomes available, future research investigating long-term drug-related adverse effects in childhood cancer survivors from both conventional cytotoxic chemotherapy and novel targeted agents may be explored. Community prescribing data from primary care records could be analysed alongside secondary care records, to investigate prescribing patterns. Second, the impact of specific treatment modalities on long-term hospitalisation risk can be answered. Third, as the objective of the study is to investigate the incidence of comorbidities in children, we used the initial admission as the primary outcome. Future studies with repeated hospitalisations as an outcome may provide information on the extended burden of comorbidity.^62^ Subsequent diagnoses of primary malignancy or cancer relapse in childhood cancer survivors can be investigated in the context of the implementation of a personalised plan for screening^63^ and cancer waiting times to determine whether survivors are subjected to a lower threshold for referral of suspected cancer. Fourth, to prevent the adverse outcome of cancer and treatment toxicity, research on the primary prevention for cancer, such as vaccines,^64^^,^^65^ could be facilitated and supported. Fifth, the mechanisms of sex disparities have yet been elucidated, and our study highlights the need for future studies to address the sex differences in disease onset and progression in children and youth. Sixth, the impact of socioeconomic deprivation on long-term morbidity in children and youths also warrants further investigation.

## Conclusion

We found that the risk of hospitalisation in all children is influenced by sex and socioeconomic deprivation. Childhood cancer survivors experienced increased risks of hospitalisation for a range of comorbidities. Population-based data provides a valuable resource to the health care system to further understand and mitigate these risks by identifying children and young adults at high risk of hospitalisation and to plan, resource and refine targeted monitoring and interventions. Children are transferred to adult services once they reach 18 but begin a pathway of transition to adult services in adolescence, which presents a valuable opportunity for dedicated service provision for morbidity surveillance and prevention that is patient-centred, risk-stratified and needs-based. Information from longitudinal data can help to optimise their future care.

## Contributors

Research question: S.C.C., A.G.L.

Funding: A.G.L.

Study design and analysis plan: S.C.C., A.G.L.

Preparation of data: S.C.C., S.M., W.H.C., A.G.L.

Statistical analysis: S.C.C.

Drafting initial and final versions of manuscript: S.C.C., A.G.L.

Critical review of early and final versions of manuscript: S.C.C., S.M., K.G., W.H.C., D.H., A.G.L.

## Data availability statement

Data may be obtained from a third party and are not publicly available. Data used in this study were accessed through NHS Digital that is subject to protocol approval and cannot directly be shared. Permission to use de-identified data from Hospital Episode Statistics for this work was granted by NHS Digital (DARS–NIC-06527). All results are reported in the manuscript and no additional data are available.

## Patient consent for publication

Not required.

## Ethics approval

This work received ethics approval from the Health Research Authority Research Ethics Committee and the London Camden & Kings Cross Committee (18/LO/0010).

## Declaration of interests

None.

## Appendix Supplementary materials

Image, application 1
