# Supplementary material for: Multimorbidity patterns and risk of hospitalisation in children: A population cohort study of 3.6 million children in England, with illustrative examples from childhood cancer survivors
Source: Lancet Reg Health Eur. 2022 Jun 30;20:100433. doi: 10.1016/j.lanepe.2022.100433 (PMC9253994; doi:10.1016/j.lanepe.2022.100433)
Supplement: Supplementary file 1 [file mmc1.docx]

Supplementary appendix for the study

**Multimorbidity patterns and risk of hospitalisation in childhood cancer survivors and children without cancer: a population cohort study of 3.6 million children and young adults in England**

Sheng-Chia Chung, Stefanie Mueller, Katherine Green, Wai Hoong Chang, Darren Hargrave, Alvina G. Lai

Table of contents

Table S1: ICD-9 and ICD-10 codes for cancer and cancer types.

Table S2: ICD-10 codes for chronic conditions.

Figure S1: Flow diagram depicting inclusion and exclusion criteria.

Section 1: Adjusted Cox regression models.

Figure S2: Graphical representation in how time-to-event would have been calculated for a typical case for cancer survivors and non-cancer survivors.

Table S3: Demographic characteristics between childhood cancer survivors and non-cancer comparisons and index cancer type in survivors.

Figure S3: Cumulative incidence of comorbid condition in the study population.

Table S4: Cumulative incidence of hospital admissions of condition categories by sex and socioeconomic deprivation status.

Figure S4: Cumulative incidence of hospitalised comorbidities by index cancer type in survivors. Each of the comorbidities are displayed as spokes on the radar plots.

Figure S5: Adjusted Cox regression analyses for risk of hospital admissions of condition categories by in cancer survivors compared with children without cancer as the reference.

Table S1: ICD-9 and ICD-10 codes for cancer and cancer types.

| 1.C00-C14 | Malignant neoplasms of lip, oral cavity and pharynx |
| --- | --- |
| 1.140-149 | Malignant Neoplasm Of Lip, Oral Cavity, And Pharynx |
| 2.C15-C26 | Malignant neoplasms of digestive organs |
| 2.150-159 | Malignant Neoplasm Of Digestive Organs And Peritoneum |
| 3.C30-C39 | Malignant neoplasms of respiratory and intrathoracic organs |
| 3.160-165 | Malignant Neoplasm Of Respiratory And Intrathoracic Organs |
| 4.C40-C41 | Malignant neoplasms of bone and articular cartilage |
| 4.C43-C44 | Melanoma and other malignant neoplasms of skin |
| 4.C45-C49 | Malignant neoplasms of mesothelial and soft tissue |
| 4.C50 | Malignant neoplasms of breast |
| 4.170-176 | Malignant Neoplasm Of Bone, Connective Tissue, Skin, And Breast |
| 5.C51-C58 | Malignant neoplasms of female genital organs |
| 5.C60-C63 | Malignant neoplasms of male genital organs |
| 5.C64-C68 | Malignant neoplasms of urinary tract |
| 5.179-189 | Malignant Neoplasm Of Genitourinary Organs |
| 6.C69-C72 | Malignant neoplasms of eye, brain and other parts of central nervous system |
| 6.190 – 192 | Malignant neoplasm of eye, brain, other and unspecified parts of nervous system |
| 7.C73-C75 | Malignant neoplasms of thyroid and other endocrine glands |
| 7.193 - | Malignant neoplasm of thyroid gland |
| 7.194 - | Malignant neoplasm of other endocrine glands and related structures |
| 8.C76-C80 | Malignant neoplasms of ill-defined, other secondary and unspecified sites |
| 8.C7A | Malignant neuroendocrine tumors |
| 8.195 - | Malignant neoplasm of other and ill-defined sites |
| 8.199 - | Malignant neoplasm without specification of site |
| 8.C7B | Secondary neuroendocrine tumors |
| 8.196 – 198 | Secondary and unspecified malignant neoplasm of lymph nodes, respiratory and digestive systems, other specified sites |
| 9.C81-C96 | Malignant neoplasms of lymphoid, hematopoietic and related tissue |
| 9.200-209 | Malignant Neoplasm Of Lymphatic And Hematopoietic Tissue |

Table S2: ICD-10 codes for chronic conditions.

| Condition | Comorbidity | ICD10 codes |
| --- | --- | --- |
| Mental health/behavioural | Substance abuse | F10,F11,F12,F13,F14,F15,F16,F17,F18,F19,K70,F55,Y47,Y49,E244,G240,G312,G405,G621,G720,G721,I426,K292,K852,K853,K860,O354,R781,R782,R783,R784,R785,Z502,Z503,Z714,Z715,Z722,Z864 |
|  | Self-harm  Other mental health problems | X chapter, Y10,Y11,Y12,Y13,Y14,Y15,Y16,Y17,Y18,Y19,Y20,Y21,Y22,Y23,Y24,Y25,Y26,Y27,Y28,Y29,Y30,Y31,Y32,Y33,Y34,Y870,Y872,Z915 |
|  |  | F00,F01,F03,F04,F05,F06,F07,F08,F09,F50, F53, F54, F59, F99, F20,F21,F22,F23,F24,F25,F26,F27,F28,F29,F30,F31,F32,F33,F34,F35,F36,F37,F38,F39,F40,F41,F42,F43,F44,F45,F46,F47,F48,F60,F61,F62,F63,F64,F65,F66,F67,F68,F69,F028,Z093,Z504,Z865,Z914 |
|  | Behavioural/developmental disorders | F70,F71,F72,F73,F74,F75,F76,F77,F78,F79,F81,F82,F83,F84,F88,F89,F90,F91,F92,F93,F94,F95,F96,F97,F98,F800,F801,F802,F808,F809 |
| Neoplasms | Neoplasms | C chapter, D00,D01,D02,D05,D06,D07,D08,D09,D12,D13,D15,D20,D32,D33,D34,D35, D37,D38,D39,D40,D41,D42,D43,D44,D45,D46,D47,D48,Z08,Z85,D141,D142,D143,D144,D630,E340,E883,G130,G131,G533,G550,G631,G73, G732,G941,M360,M361,M495,M820,M906,M907,N081,N161,Y431,Y432,Y433, Y842,Z510,Z511,Z512,Z541,Z542,Z860,Z923 |
| Immunological/Blood disorders | Immunological disorders | D80,D81,D82,D83,D84,G532,Q980 |
|  | Anaemia and other blood disorders | D50,D58,D64,D66,D67,D69,D70,D71,D72,D73,D74,D75,D76,D560,D561,D562,D564,D568,D569,D570,D571,D572,D578,D610,D619,D680,D681,D682,D684,D685,D686,D687,D688,D689,M362,M363,M364,M904,N082,Z862 |
| Chronic infections | Tuberculosis  Other infection | A15,A16,A17,A18,A19,E350,K230,K673,K930,M011,M490,P370 |
|  |  | A50,A81,B18,B45,B46,B55,B59,B67,B69,B73,B74,B90,B91,B92,B93,B94,M00 ,B371,B375,B376,B377,B381,B391,B401,B440,B447,B487,B500,B508,B510,B518,B528,B520,B572,B573,B574,B575,B580,B787,F021,K231,K931, N330,P350,P351,P352,P358,P359,P371 |
| Respiratory | Asthma and chronic lower respiratory disease | J41,J42,J43,J44,J45,J46,J47 |
|  | Respiratory injuries  Other respiratory | S17,S27,S28,T27,T914 |
|  |  | J60,J61,J62,J63,J64,J65,J66,J67,J68,J69,J70, J80,J81,J82,J83,J84,J85,J86, J98, P27,G473,J961,Y556,Z430,Z930,Z942 |
| Metabolic/endocrine/digestive/renal/genitourinary | Diabetes | E10,E12,E13,E14,G590,G632,I792,M142,N083,Y423 |
|  | Metabolic | D55,E70,E71,E72,E74,E75,E76,E77,E78,E83,E85,E791,E792,E793,E794,E795,E796,E797,E798,E799,E800,E801,E802,E803,E805,E807,E880,E881,E882,E888,E889,G736,L990,M144,M143,N163 |
|  | Digestive | K20,K22,K25,K26,K27,K28,K31,K50,K51,K52,K55,K57,K66,K72,K73,K74,K75,K76,K80,K81,K82,K83,K90,K210,K238,K290,K291,K293,K294,K295,K296,K297,K298,K299,K592,K630,K631,K632,K633,K850,K851,K858,K859,K861,K862,K863,K864,K865,K866,K867,K868,K869,K870,M074,M075,M091,M092,T864,Z432,Z434,Z465,Z903,Z904,Z932,Z933,Z934,Z935 |
|  | Renal/ genitourinary | N00,N01,N02,N03,N04,N05,N07,N11,N12,N13,N14,N15,N18,N19,N20,N21,N22,N23, N25,N26,N28,N29,N31,N32,N35,N36,N40,N41,N42,N70,N71,N72,N73,N74,N80,N81,N82,N85,N86,N87,N88,Z49,D638,G638,G998,I688,M908,N084,N160,N162,N164,N165,N168,N338,N391,N393,N394,P960,T824,T831,T832,T834,T835,T836,T837,T838,T839,T855,T861,Y602,Y612,Y622,Y841,Z936,Z940,Z992 |
|  | Metabolic/GI injuries  Metabolic/GI other/unspecific | S36,S37,S38,T28,S396,S397,T065,T915 |
|  |  | E66,N92,G633,G990,M145,Z863,Z938 |
| Musculoskeletal/skin | Musculoskeletal/connective tissue | M05,M06,M08,M10,M11,M12,M13,M30,M31,M32,M33,M34,M35,M40,M41,M42,M43,M45, M46,M47,M48,M50,M51,M52,M53,M54,M60,M61,M62,M85, M89,M91,M92,M93,M94 ,G551,G552,G553,G635,G636,G737,J990,J991,L620,M070,M071,M072,M073,M076,M098,M140,M146,M148,M638,M801,M802,M803,M804,M805,M806,M807,M808,M809,M811,M812,M813,M814,M815,M816,M817,M818,M819,M821,M828,M840,M841,M842,M848,M849,M863,M864,M865,M866,M900,N085,Y454 |
|  | Skeletal injuries/amputations | S13,S23,S32,S33,S77,S78,S87,S88,S97,T02,T04,T05,S220,S221,S222,S225,S683,S684,S688,S980,S982,S983,S984,T203,T207,T213,T217,T223,T227,T232,T233,T236,T237,T243,T247,T252,T253,T256,T257,T293,T297,T303,T307,T312,T313,T314,T315,T316,T317,T318,T319,T322,T323,T324,T325,T326,T327,T328,T329,T873,T874,T875,T876,T912,T918,T926,T931,T934,T936,T940,T941,T950,T951,T954,T958,T959,Y835,Z891,Z892,Z895,Z896,Z898,Z971 |
|  | Chronic skin disorders | L10,L12,L13,L14,L28,L40,L41,L42,L43,L44,L45,L57,L59,L87,L88,L90,L92,L95, L93,Q80,Q81,L110,L118,L119,L581,L985,M090,Q870,Q871,Q872,Q873,Q874,Q875,Q894 |
| Neurological | Epilepsy | G41,F803,G400,G401,G402,G403,G404,G406,G407,G408,G409,R568,Y460,Y461,Y462,Y463,Y464,Y465,Y466 |
|  | Injuries of brain,nerves,eyes or ears | S05,S06,S07,S08,S12,S14,S24,S34,S44,S54,S64,S74,S84,S94,T26,T060,T061,T062,T904,T905,T911,T913,T924 |
|  | Chronic eye conditions | H17,H18,H33,H34,H35,H40,H21,H26,H27,H31,H43,H44,H47,H051,H052,H053,H054,H055,H056,H0527,H058,H059,H133,H193,H198,H280,H281,H282,H328,H420,H540,H541,H542,H544,T852,T853,Z442 |
|  | Chronic ear conditions | H80,H91,H602,H652,H653,H654,H661,H662,H663,H690,H701,H731,H740,H741,H742,H743,H750,H810,H814,H830,H832,H900,H903,H905,H906,Z453 |
|  | Other neurological | G00,G01,G02,G03,G04,G05,G06,G07,G08,G09,G10,G11,G12,G14,G20,G21,G22,G23, G24,G25,G26,G27,G28,G29,G30,G31,G32,G33,G34,G35,G36,G37,G43,G44,G45,G46,G50,G51,G52,G54,G56,G57,G58,G60,G61,G64,G70,G71,G90,G91,G92,G93,G95,G96,G98,I60,I61,I62,I63,I64,I65,I66,I67,I69,F022,F023,G138,G241,G242,G243,G244,G245,G246,G247,G248,G249,G310,G311,G318,G319,G470,G471,G472,G474,G475,G476,G477,G478,G479,G530,G531,G538,G558,G598,G620,G622,G623,G624,G625,G626,G627,G628,G629,G722,G723,G724,G725,G726,G727,G728,G729,G730,G733,G942,G948,G991,G992,I680,I682,I720,I725,T850,T851,Y467,Y468,Z982 |
| Cardiovascular | Other cardiovascular | I00,I01,I02,I03,I04,I05,I06,I07,I08,I09,I10,I11,I12,I13,I14,I15,I16,I17,I18,I19,I20,I21,I22,I23,I24,I25,I26,I27,I28,I31,I32,I33,I34,I35,I36,I37,I38,I39,I50,I51,I70I41,I46,I47,I48,I49,I71,I73,I74,I75,I76,I77,I81,I82,I98,I99,Q27,Q28,S26,Z95,M036,N088,I420,I421,I422,I423,I424,I425,I427,I428,I429,I430,I431,I432,I433,I434,I435,I436,I437,I438,I441,I442,I443,I444,I445,I446,I447,I451,I452,I453,I454,I455,I456,I457,I458,I459,I528,I721,I722,I723,I724,I728,I729,I790,I791,I798,T820,T821,T822,T823,T825,T826,T827,T828,T829,T862,Y605,Y615,Y625,Y840,Z450,Z500,Z941 |
| Fever/acute respiratory/sepsis | fever | R509,R508,R502 |
|  | acute respiratory | J068,J069,J101,J111,J205,J210,J22X,J393,J398,J399,J958,J959,J960,P228,P288,R048,R049,R840,R845,R846,R847,R848,R849,U049 |
|  | sepsis | A021,A227,A400,A401,A402,A403,A408,A409,A410,A411,A412,A413,A414,A415,A418,A419,A427,O85X,P360,P361,P362,P363,P364,P365,P368,P369 |

Adapted from Overview of child deaths in the four UK countries. 2013 Royal College of Paediatrics and Child Health <https://www.rcpch.ac.uk/sites/default/files/CHR-UK_-_Retrospective_Epidemiological_Review_of_All-cause_Mortality_in_CYP.pdf>

Figure S1: Flow diagram depicting inclusion and exclusion criteria.
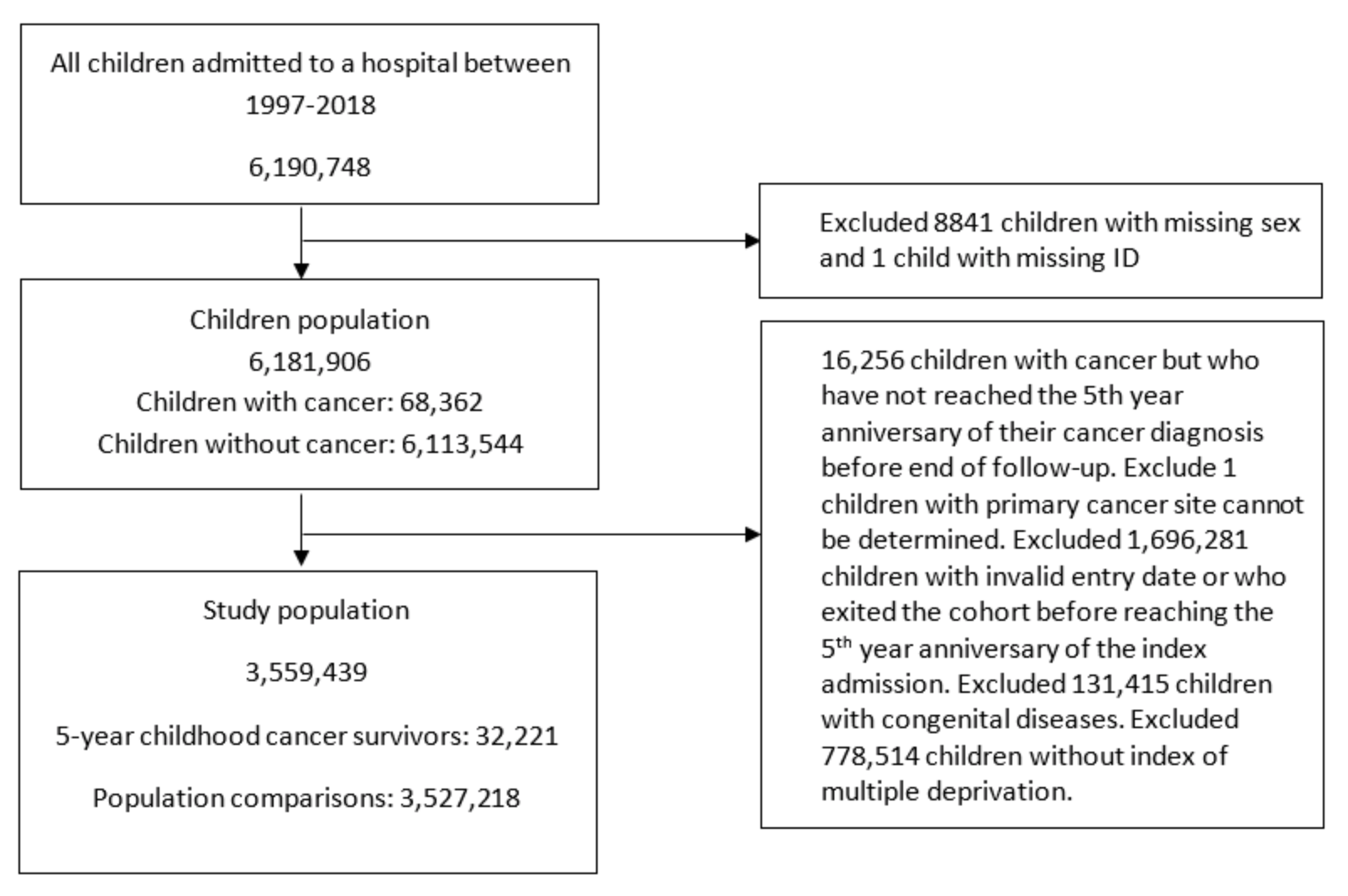


Figure S2: Graphical representation in how time-to-event would have been calculated for a typical case for cancer survivors and non-cancer survivors.


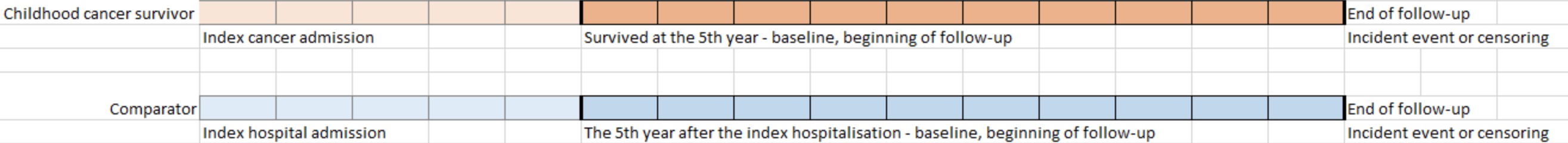


*One block represent 1 year.

Section 1: Adjusted Cox regression models.

The time to event model was based on population at risk of the modelled condition/comorbidity. For example, modelling time incident condition A will be construct among the participants free from condition A at baseline. A death occurred during the study period was considered as a competing risk to the outcome of interest (i.e. incident comorbid condition/comorbidity). To adjust for competing risk in the survival analyses, we applied Cox regression model to estimate cause-specific hazard where competing events are treated as censored observations.^1^ The Cox regression models in the study were also adjusted by age at baseline (continuous variable), binary sex (girl=1, boy=0) and categorical Index of multiple deprivation quintile: 1^st^ (least deprived), 2^nd^ , 3^rd^ and 4^th^ quintile comparing to the reference group of the 5^th^ quintile (most deprived areas).

^1^So Y, Lin G, Johnston G. Using the PHREG procedure to analyze competing-risks data. SAS Global Forum 2014 Mar 23 (Vol. 2014, pp. 23-26).

Table S3: Demographic characteristics between childhood cancer survivors and non-cancer comparisons and initial cancer type in survivors.

|  | All childhood cancer survivors | Population comparisons |
| --- | --- | --- |
| N | 32221 | 3527218 |
| Age, years, mean (SD) | 14.3 (6.0) | 11.2 (6.3) |
| Girl. % | 14595 (45.3) | 1684052 (47.7) |
| Index of multiple deprivation Quintile | | |
| 1st (least deprived), % | 6311 (19.6) | 555265 (15.7) |
| 2nd, % | 6024 (18.7) | 577188 (16.4) |
| 3rd, % | 6589 (20.5) | 758560 (21.5) |
| 4th, % | 6515 (20.2) | 750274 (21.3) |
| 5th (most deprived), % | 6773 (21.0) | 885931 (25.1) |


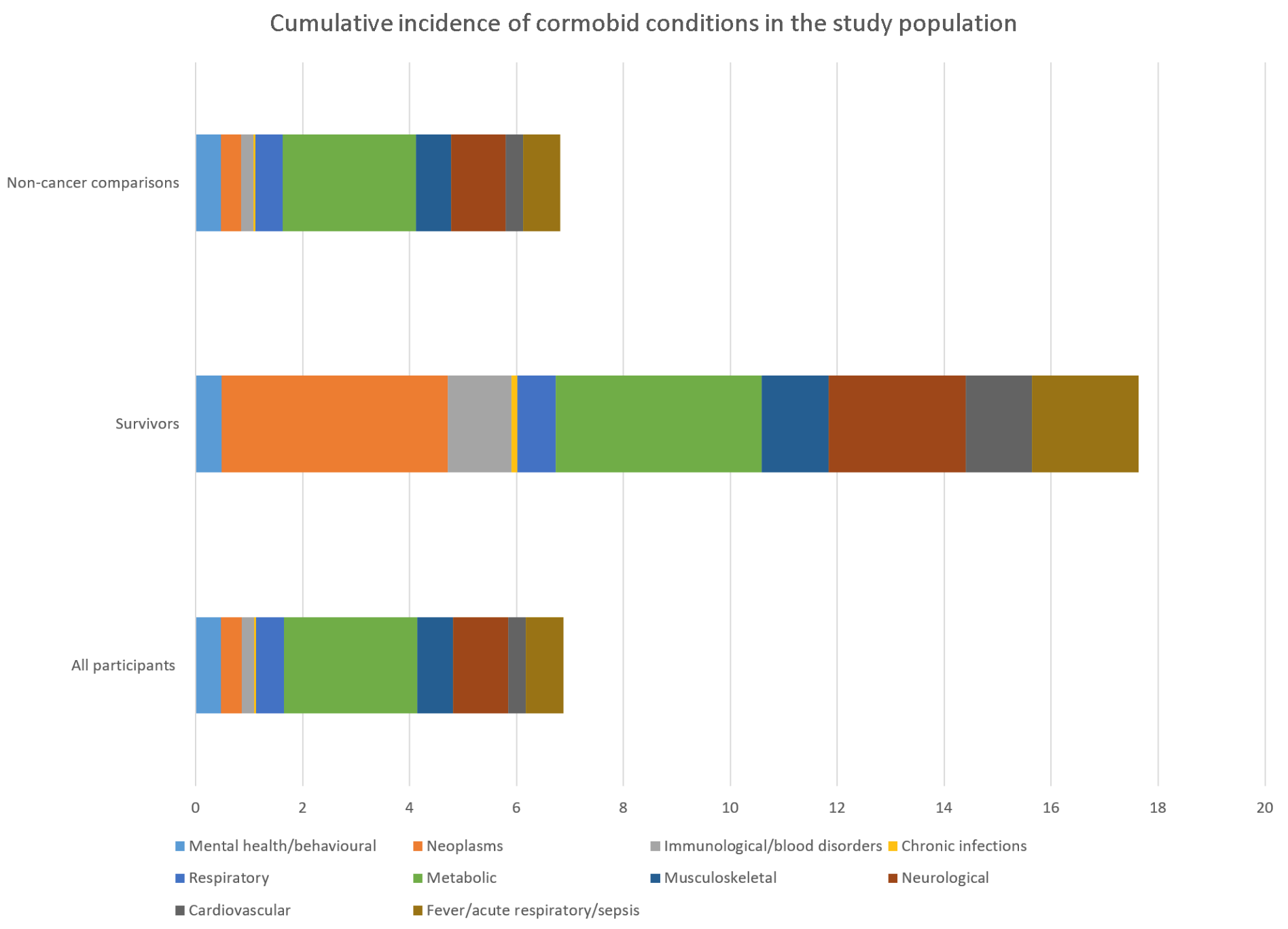


Figure S3: Cumulative incidence of comorbid condition in the study population.

Table S4: Cumulative incidence of hospital admissions of condition categories by sex and socioeconomic deprivation status.


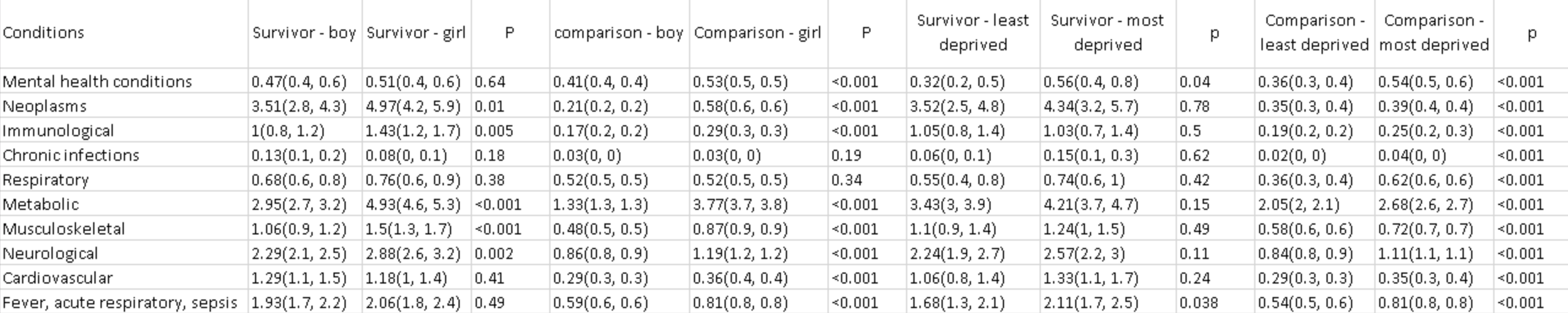


* p-value for the Wald chi-square statistics.


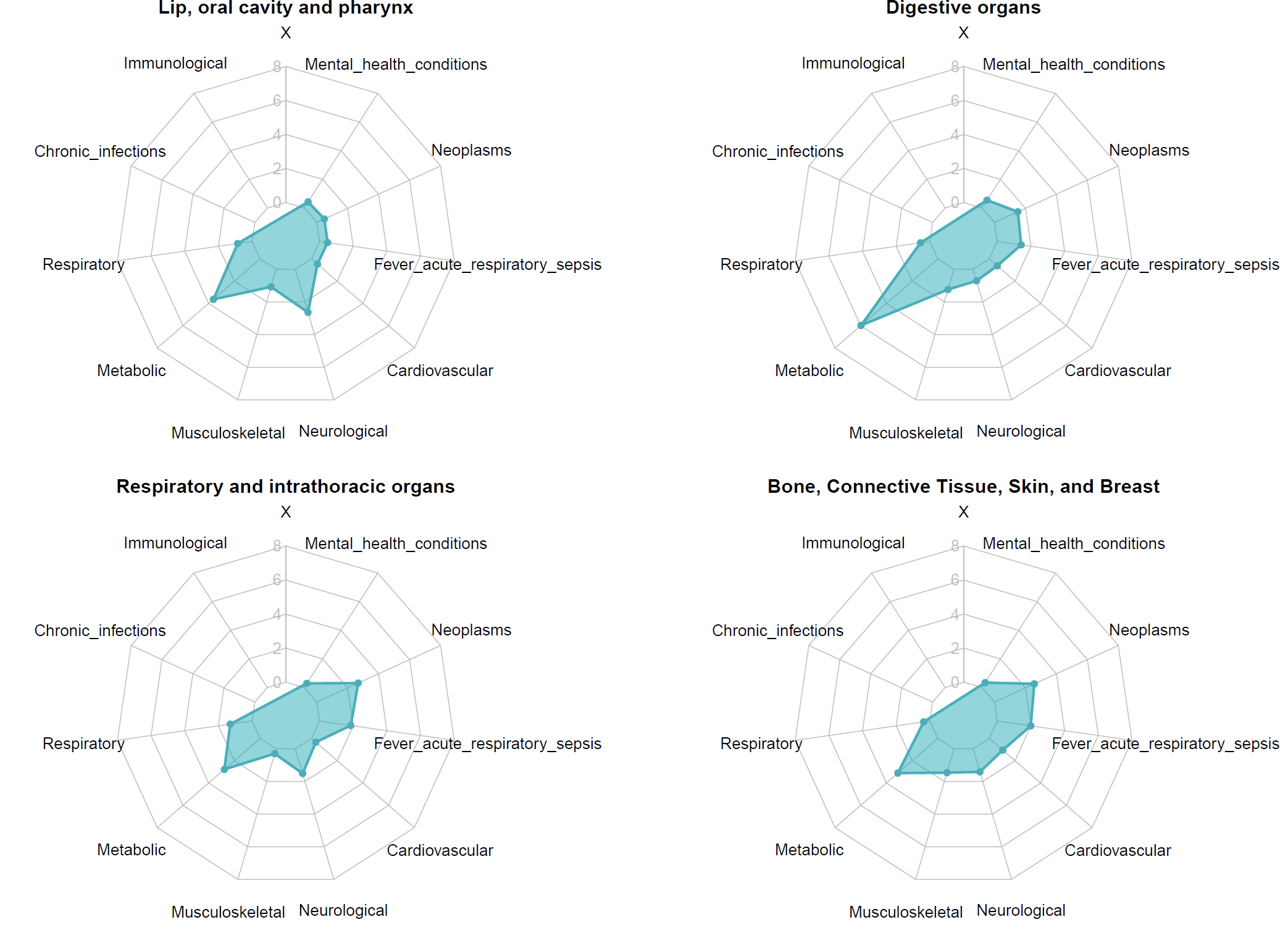


Figure S4: Cumulative incidence of hospitalised comorbidities by index cancer type in survivors. Each of the comorbidities are displayed as spokes on the radar plots.


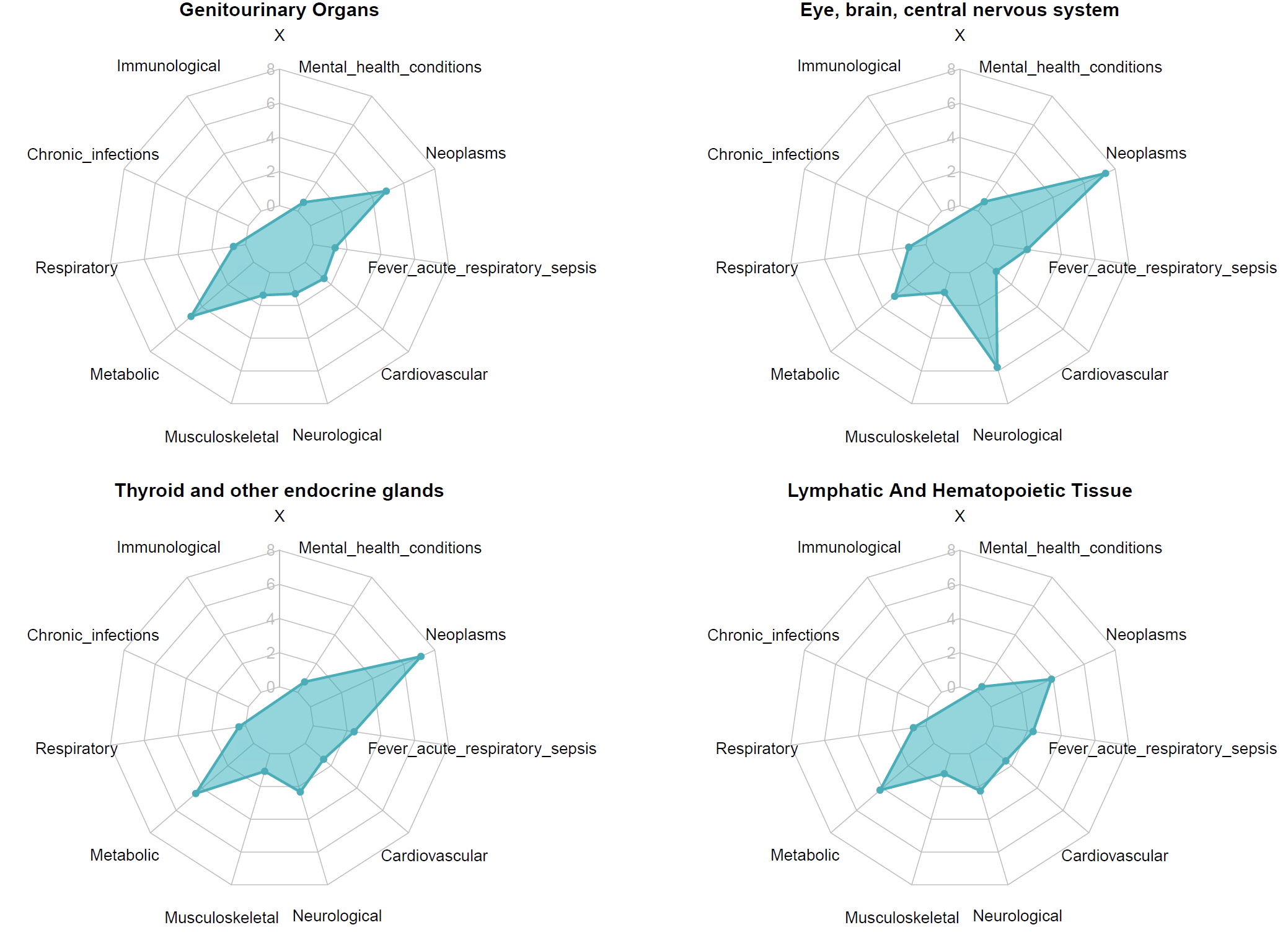


Figure S4: Cumulative incidence of hospitalised comorbidities by index cancer type in survivors. Each of the comorbidities are displayed as spokes on the radar plots (continue.)


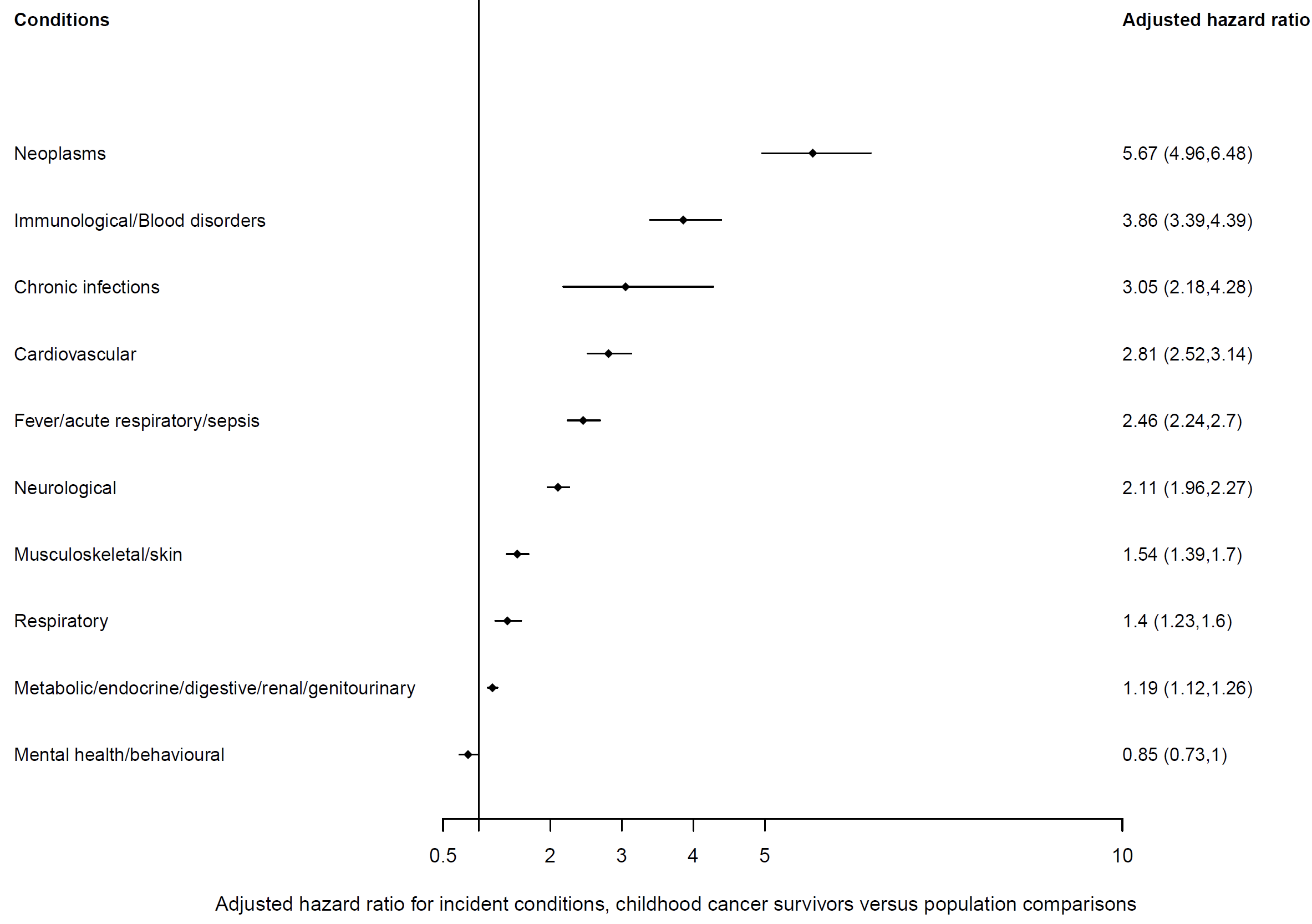


Figure S5: Adjusted Cox regression analyses for risk of hospital admissions of condition categories by in cancer survivors compared with children without cancer as the reference.
